# Transfer Learning for Soil Spectroscopy Based on Convolutional Neural Networks and Its Application in Soil Clay Content Mapping Using Hyperspectral Imagery

**DOI:** 10.3390/s18093169

**Published:** 2018-09-19

**Authors:** Lanfa Liu, Min Ji, Manfred Buchroithner

**Affiliations:** Institute for Cartography, TU Dresden, 01062 Dresden, Germany; Lanfa.Liu@outlook.com (L.L.); Manfred.Buchroithner@tu-dresden.de (M.B.)

**Keywords:** transfer learning, deep learning, CNNs, hyperspectral imagery, soil spectroscopy

## Abstract

Soil spectra are often measured in the laboratory, and there is an increasing number of large-scale soil spectral libraries establishing across the world. However, calibration models developed from soil libraries are difficult to apply to spectral data acquired from the field or space. Transfer learning has the potential to bridge the gap and make the calibration model transferrable from one sensor to another. The objective of this study is to explore the potential of transfer learning for soil spectroscopy and its performance on soil clay content estimation using hyperspectral data. First, a one-dimensional convolutional neural network (1D-CNN) is used on Land Use/Land Cover Area Frame Survey (LUCAS) mineral soils. To evaluate whether the pre-trained 1D-CNN model was transferrable, LUCAS organic soils were used to fine-tune and validate the model. The fine-tuned model achieved a good accuracy (coefficient of determination (*R*^2^) *=* 0.756, root-mean-square error (*RMSE*) *=* 7.07 and ratio of percent deviation (*RPD*) *=* 2.26) for the estimation of clay content. Spectral index, as suggested as a simple transferrable feature, was also explored on LUCAS data, but did not performed well on the estimation of clay content. Then, the pre-trained 1D-CNN model was further fine-tuned by field samples collect in the study area with spectra extracted from HyMap imagery, achieved an accuracy of *R*^2^ *=* 0.601, *RMSE =* 8.62 and *RPD =* 1.54. Finally, the soil clay map was generated with the fine-tuned 1D-CNN model and hyperspectral data.

## 1. Introduction

Soil spectroscopy has the capability to rapidly and non-destructively analyse soil properties by taking advantage of visible near-infrared shortwave infrared (Vis–NIR–SWIR) spectral information [1,2,3,4,5]. There are numeral studies related to the reliable estimation of soil properties using prepared soil samples and measured spectral data [6,7,8,9]. Although the relationship between soil properties and the corresponding spectra is complex and soil spectroscopy is less accurate than wet chemistry, it still achieved great success in laboratory studies, which naturally leads to the exploration of imaging spectroscopy (IS) for characterising soil properties at large scales. It not only has the capability of obtaining spectral information at several hundred spectral bands as laboratory spectroscopy does, but also provides a spatial view, which cannot be achieved by laboratory techniques [10]. IS technology provides the opportunity to map various soil properties at regional and global scales at comparatively low costs.

The spectral features and quantitative estimation of clay content in soil have been explored in previous studies [11,12,13,14]. In Reference [15], the clay content was demonstrated to be strongly correlated with the clay minerals in soil and the principal characteristic bands were related to the lattice hydroxyl groups. Clay minerals have characteristic absorptions near 1400 nm and 2200 nm [3]. The absorption feature near 1400 nm is due to overtones of the O-H stretch vibration, while the absorption near 2200 nm is due to Al-OH bend plus O-H stretch combinations. A clay spectral index was further proposed using the absorption feature near 2200–2300 nm in Reference [16]. The performance of spectra measured in the well-controlled laboratory and acquired from IS sensors has been assessed by many case studies for soil property estimation [17,18,19,20,21]. The accuracy using imaging spectroscopy is comparatively lower than the result obtained from laboratory spectroscopy, as the application of imaging spectroscopy in the assessment of topsoil properties is constrained by many factors, such as the low signal-to-noise ratio, atmosphere attenuation, revisiting time, sensor radiometric and spatial resolutions, vegetation coverage and Bidirectional Reflectance Distribution Functional (BRDF) [22,23]. The distance from the sensor to soil samples is often between 1 and 140 cm in the laboratory [24] while the IS has a much far distance, like the satellite-borne Hyperion hyperspectral flying at 705 km altitude [25]. The calibration and performance of different sensors are also determinants of the quality of measured spectra. Soil samples are often illuminated with 1–6 light sources [24] in the laboratory while air- or satellite-borne hyperspectral imagery is obtained under solar illumination. Besides, laboratory soil samples are dried, crushed and sieved while imaging targets in the field are natural surfaces with heterogeneous surface temperatures, moisture levels and roughness [26]. Moisture effects on the soil spectral reflectance have been studied extensively [1,27]. The overall reflectance generally decreased, with an increasing amount of moisture. Furthermore, the absorption by water in the SWIR region impacted clay-associated absorption features [28]. These factors lead to spectral differences between laboratory and remotely sensed data.

Soil spectral libraries can be used as a reference for retrieving soil attributes by reflectance spectroscopy. Calibrations are not reliable for soils not represented in the soil spectral library, hence there is a need for building libraries representative of the soil diversity [29,30] and an increasing number of large-scale soil spectral libraries established at national, continental and even global levels. As a key innovation, near and mid-infrared spectroscopy are used for soil analysis in the collaborative Africa Soil Information Service (AfSIS) project, which covers an area, including about 17.5 million km^2^ of continental sub-Saharan Africa (SSA) and almost 0.6 million km^2^ of Madagascar [31]. In the first period (2009–2012) of Land Use/Land Cover Area Frame Survey (LUCAS), which is an extensive topsoil survey that is carried out across the European Union to derive policy-relevant statistics on the effect of land management on soil characteristics, soil spectra of about 20,000 topsoil samples were acquired in the range of 400–2500 nm and extensively studied [7,32,33,34,35,36]. A new LUCAS sampling campaign will be undertaken in 2018 [37]. A voluntary collaborative project was started in 2008 to develop a global library of soil spectra, and 23,631 soil spectra have been contributed to the global database by around 45 soil scientists and researchers from 35 institutions [8]. In addition, there are a number of national and regional soil spectral libraries have been established, such as the ones for Australia [38], Czech Republic [39], Brazil [24] and China [40]. A soil library typically contains soil attributes as done by wet chemistry standard methods and reflectance spectra acquired under a routine protocol and spectrometer. However, there is still lack of protocols for soil spectral measurements. The internal soil standard (ISS) concept is proposed to make soil spectra from different libraries sharable by minimising the systematic effects [41,42].

Large soil spectral libraries should help to reduce or even save the need to collect and analyse new samples for site-specific calibrations to estimate soil properties, and it could be a strong base for hyperspectral remote sensing of soils from space [4]. The laboratory soil spectra may enable appropriate validation of the reflectance information acquired from IS sensors. However, there are still few studies integrating IS with laboratory studies [26,43,44,45,46]. In Reference [23], it is pointed out that calibration models developed from laboratory processed samples cannot be utilised for field spectroscopy, due to the influence of external environmental factors (such as soil moisture, soil roughness, atmospheric effect and vegetation coverage). Furthermore, spectroscopic models achieved by common calibration methods are usually not transferrable. An important drawback of Partial least squares (PLS) regression is the complexity of the transfer of spectroscopic models from one sensor to another [43,47]. When samples to be predicted are far away from the spectral library, the regression algorithm is prone to fail in producing reliable model soil predictions [33].

It is suggested that spectral indices may provide an alternative method to PLS regression for quantifying soil contents in situations where calibration models should be transferred between different spectrophotometers [48]. The soil organic carbon (SOC) estimation was carried out using simple and multiple linear regression techniques based on image reflectance values and spectral indices, which confirmed that spectral indices have potential to be transferred among airborne and satellite hyperspectral sensors [49]. Spectral indices can be viewed as simple transferable features developed by combining surface reflectance at two or more wavelengths that indicate relative abundance of features of interest. A number of soil spectral indices have been proposed for the estimation of SOC, soil salinity, soil clay and iron [16,48,50,51]. Transfer learning aims to propagate the knowledge from a source domain to a target domain [52]. Therefore, it has the potential to make calibration models transferable from one sensor to another. Transfer learning with the pre-trained convolutional neural network (CNN) model has been proposed for remote sensing. CNNs can learn representative and discriminative features in a hierarchical manner from the data [53], and have recently been widely used in various remote sensing data analysis tasks, such as classification, segmentation, object detection, image registration, and change detection [54,55,56,57,58]. A comprehensive review and list of resources using CNNs for remotely sensed data can be found in Reference [59]. The transferability of the natural image features from the pre-trained CNN models has been explored to the limited amount of high-resolution remote sensing scene datasets with the feature coding methods [60]. The advantage of adopting pre-trained CNN models is the effective extensible properties for dealing with the high-resolution remote sensing imagery scenes with limited labelling. A transfer learning method with fully pre-trained CNNs (CNN-FT-Full) was proposed to overcome the separation of asynchrony of different parts of the transferred CNNs during the learning process, and it performed well on land-use classification with high-resolution remote sensing images [61]. In Reference [62], transfer learning was proposed to transfer knowledge learned from a large amount of unlabelled SAR scene data (50,000 image patches extracted from TerraSAR-X scene images) to SAR target recognition tasks. However, there are still few studies using pre-trained CNN models in soil spectroscopy.

The objective of this study is to explore the potential of transfer learning for soil clay mapping using hyperspectral imagery and a pre-trained CNN model developed from a large number of spectra measured in the laboratory. Descriptions of laboratory and airborne spectral data are given in Section 2.1. The proposed workflow and model performance metrics are presented in Section 2.2. The results of the calibration and validation for soil clay content retrieval using laboratory-derived spectral library and the transferability for airborne spectral data are presented and subsequently discussed in Section 3. Conclusions are given in Section 4.

## 2. Materials and Methods

### 2.1. Datasets

#### 2.1.1. The LUCAS Soil Spectral Library

**Figure 1:**
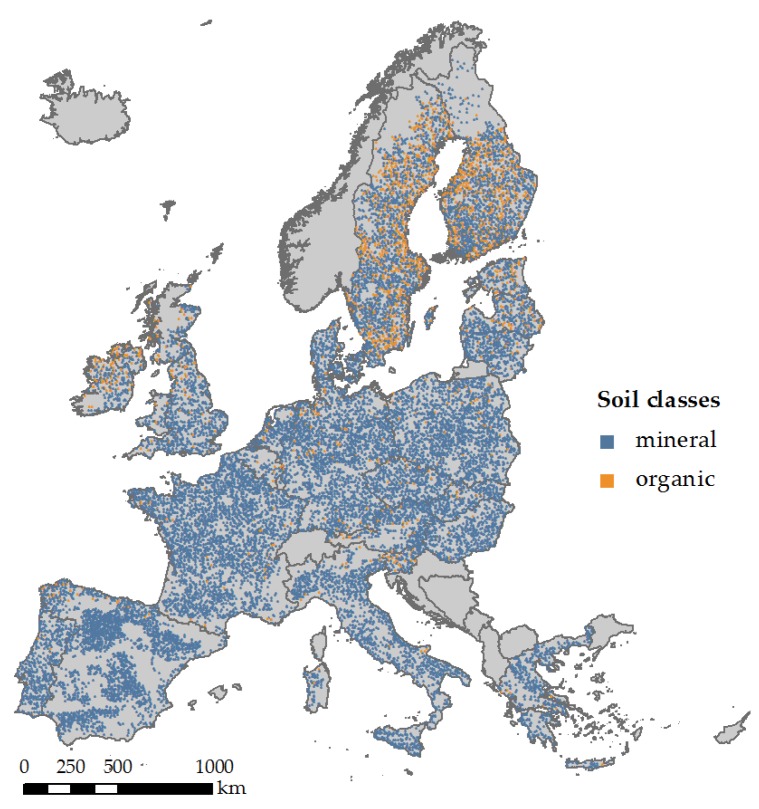
Distribution of mineral and organic soils from Land Use/Land Cover Area Frame Survey (LUCAS) soil spectral library.

The first dataset utilised for developing and evaluating the pre-trained one-dimensional convolutional neural network (1D-CNN) model is LUCAS soil spectral library, which contains approximately 20,000 geo-referenced soil samples that collected and analysed across Europe [63,64]. A standardised sampling procedure was adopted to collect around 0.5 kg of topsoil (0–20 cm) in the field. The distribution of LUCAS soil samples can be seen in Figure 1. Soil samples can be divided into mineral and organic soils according to [35]. Soil spectra were measured using a FOSS XDS Rapid Content Analyser, operating in the 400–2500 nm wavelength range, with 0.5 nm spectral resolution. Pre-processed included transformation of absorbance (A) spectra into reflectance (1/10^A^) spectra and Savitzky-Golay Filter with a window size of 50, second order polynomial. The laboratory spectral data were resampled to be in consistent with bands of the HyMap imagery, so that the model developed using LUCAS data can also accept HyMap data as inputs.

#### 2.1.2. Cabo de Gata-Nijar Hyperspectral Imagery

**Figure 2:**
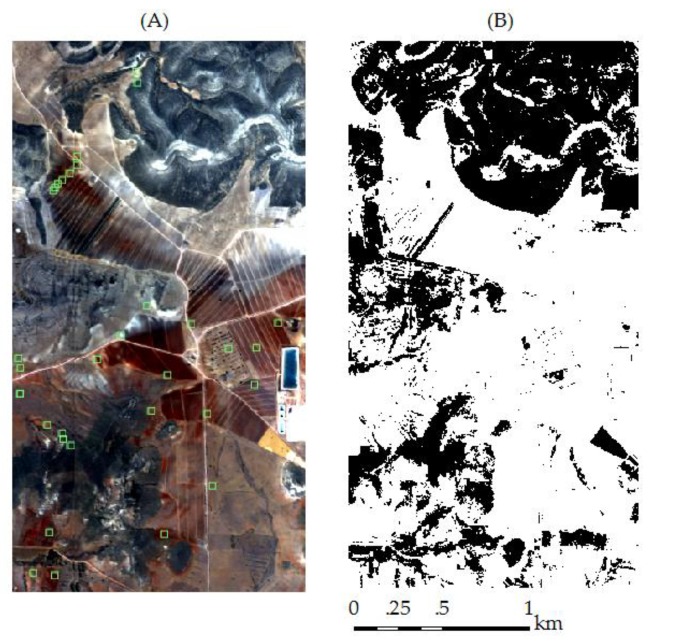
HyMap imagery (**A**) and soil mask (**B**) in the study area Cabo de Gata-Nijar. The locations of field samples were shown in green squares.

The second dataset is the hyperspectral imagery acquired in the Natural Park Cabo de Gata-Níjar in the Almeria province of southeastern Spain. Our study focuses on a small area at Cortijo del Fraile, which is an agricultural area in the middle of the park with mostly bare fields at the time of the overflight. In June 2005, airborne hyperspectral data were obtained over the small area with the HyMap sensor (Baulkham Hills, NSW, Australia) [65]. It provided spectral images after processing to geocoded reflectance covering the spectral range of 400 to 2450 nm with a spectral resolution of 12 to 17 nm [43]. The average flight altitude of 2645 m above sea level resulted in a spatial resolution of 5 m. The raw HyMap data were corrected to at-sensor-radiance based on calibration coefficients obtained during laboratory calibration by HyVista. The atmospheric correction was performed with ATCOR4 software. A mask was applied to the airborne data to keep pixels of bare soil surface only. The soil mask (Figure 2B)was created following the approach provided by ENSOMAP software, which is an open source tool for quantitative soil properties mapping based on hyperspectral imagery [66].

32 soil samples were randomly taken from the upper soil surface (0–2 cm) in the study area and the corresponding locations can be seen in Figure 2A. Samples were air dried and passed through a 2 mm sieve before laboratory analysis. The particle size distribution was determined by wet sieving the sand fraction and using the pipette method for silt and clay fractions after the removal of organic matter with H_2_O_2_ and dispersion with Na-hexametaphosphate. The clay content values of field samples vary between 8.4% and 63.4%. Collected soil samples were randomly divided into two subsets with a ratio of 1:1 to calibrate and validate the fine-tuned model. A brief statistical summary can be seen in Table 1.

### 2.2. Methods

**Figure 3:**
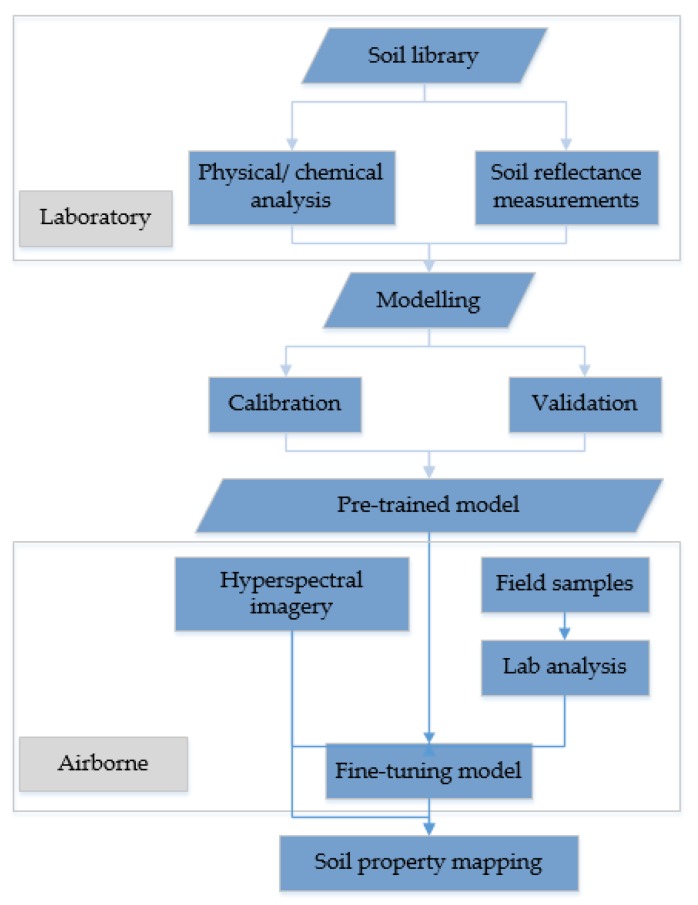
Schematic diagram of proposed workflow on transfer learning for soil property mapping.

The proposed workflow was shown in Figure 3. Spectral measurements of soil samples were acquired in the well-controlled laboratory and the corresponding soil properties were also retrieved by conventional chemical/physical analysis. A 1D-CNN model as mentioned before was developed based on the soil spectral library and will be used as the base model for further analysis. Sixteen field samples collected in the study area were used to fine-tune the pre-trained 1D-CNN model and the others 16 were for the independent validation. It is pointed out that normalized spectral indices have the potential to be transferred between sensors. Therefore, a spectral index for soil clay is also explored on the large-scale soil spectral library.

#### 2.2.1. Convolutional Neural Networks

**Figure 4:**
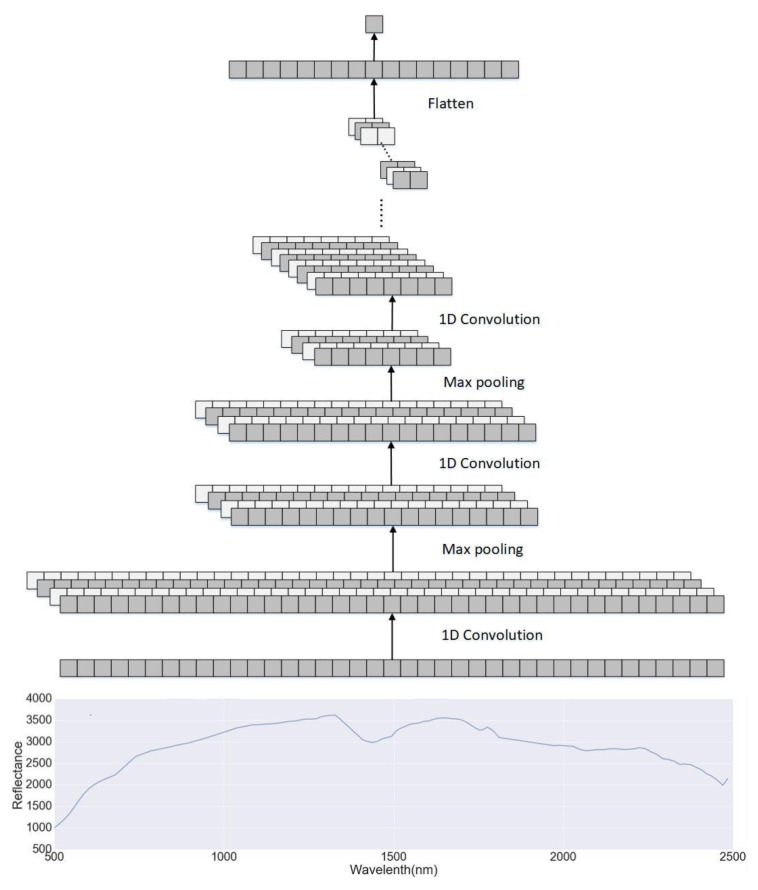
The architecture of the convolutional neural network (CNN) for soil property estimation.

The CNN is composed of multiple feature generation stages, each of which includes a convolutional layer, a nonlinearity layer and a pooling layer. After several feature generation stages, the CNN is often followed by one or more fully-connected layers and a final classifier layer for classification tasks. In this study, we adopt the CNN for the estimation of soil clay content, which is continuous data instead of categorical data. For example, the clay content values for LUCAS mineral soils range from 0.0 to 79.0%. Therefore, we use a regression layer to replace the final classifier layer. The architecture can be seen in Figure 4.

A soil spectrum can be regarded as a 2D image whose height is equal to 1 [67]. Therefore, the size of input layer can be viewed as n×1, and n is the number of bands. Each convolutional layer contains a number of 1D filter kernels with the size of k×1, which generate feature maps when applied to the input spectral data. The number of layers, the kernel size and the number of kernels in the convolutional layers are hyperparameters that set manually. In this study, we use four convolutional layers and the number of filter kernel was set to (32,32,64,64). The size of filter kernel is 3. The weights in the kernels are learnt using the back propagation (BP) algorithm with labelled training dataset. The main benefit is that feature maps used in the classification or regression are learnt from data without any manual feature extraction [68].

#### 2.2.2. Transfer Learning Based on the Pre-Trained 1D-CNN Model

It is pointed out that there are two ways to apply transfer learning with deep networks [69]. One is to take advantage of the pre-trained neural network model with the learned weights to acquire features that would be subsequently used in the new problem. Feature generation layers, prior to the last fully-connected layer, are frozen and the outputs of the CNN constitute learnt features. Another option is to fine-tune the pre-trained network weights by training the network with the new available data. As we are trying to make model transferrable between different sensors, the second method was adopted to fine-tune the whole pre-trained CNN model.

The LUCAS data is classified into two categories: Mineral and organic soils. We first use mineral soils to build a CNN model, since the CNNs typically have a large number of parameters and require a significant amount of training data. We use the spectra extracted from hyperspectral data at the locations of field samples and the corresponding soil clay content values to fine-tune the pre-trained CNN model. Finally, the fine-tuned model is applied to the whole hyperspectral image so as to obtain the soil clay content map in the study area.

#### 2.2.3. Spectral Index for Soil Clay Content

Clay minerals are characterised by absorption features near 2200–2300 nm. The location of the clay absorption peak was identified at 2209 nm with the following two wavelengths representing the shoulders of the absorption peak: 2133 nm and 2225 nm. Using these wavelengths, a short-wave infrared fine particle index (SWIR FI), as shown in Equation (3), was proposed in Reference [16] and implemented in ENSOMAP software.

(1) $$\;\mathrm{SWIR}\;\mathrm{FI}=\;\frac{{\left(b2133\;\mathrm{nm}\right)}^{2}}{b2225\;\mathrm{nm}\;\times {\left(b2209\;\mathrm{nm}\right)}^{3}}\;$$

### 2.3. Assessment

The performance of calibration models for soil clay content was assessed by root-mean-square error (*RMSE*), coefficient of determination (R^2^) and the ratio of percent deviation (*RPD*), which were calculated by the following equations:

(2) $$\;R^{2}=\frac{\sum_{i=1}^{n}{\left({\hat{y}}_{i}-\bar{y}\right)}^{2}}{\sum_{i=1}^{n}{\left(y_{i}-\bar{y}\right)}^{2}},\;$$

(3) $$\;RMSE=\sqrt{\frac{1}{n}\sum_{i=1}^{n}{\left({\hat{y}}_{i}-y_{i}\right)}^{2}},\;$$

(4) $$\;RPD=\;\frac{SD}{RMSE},\;$$

where, *n* means the number of validation samples, *y* is the measured value, $\bar{y}$ is the mean of the measured value, and $\hat{y}$ is the predicted value of soil clay content. *RPD* denotes the ratio of the standard deviation (*SD*) of the calibration data to the *RMSE* of the validation data.

## 3. Results and Discussion

### 3.1. Interpretation of Mineral and Organic Soils from LUCAS Dataset

**Figure 5:**
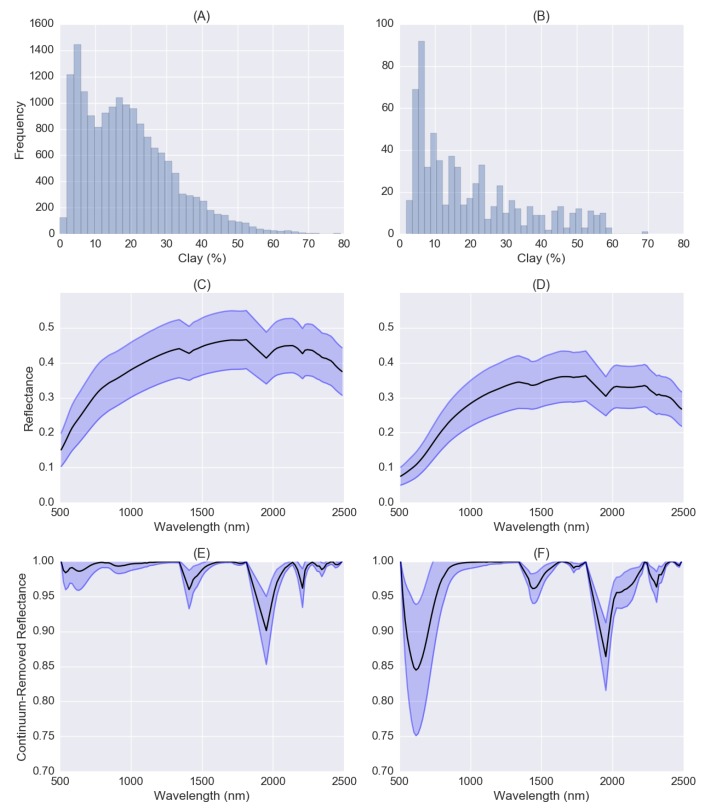
(**A**,**B**) Histograms of soil clay content distribution of mineral and organic soils; (**C**,**D**) mean soil reflectance spectra and standard deviations for mineral and organic soils; (**E**,**F**) mean soil continuum-removal spectra and standard deviations for mineral and organic soils. Values are given in reflectance (**C**,**D**) and normalised continuum-removal values (**E**,**F**).

The LUCAS dataset contains about 16,000 soil samples classified as mineral soils that were used to train the one-dimensional CNN model. About 660 organic soil samples containing clay information were used to test if CNN model developed by mineral soils is transferrable for organic soils. The histograms of soil clay content distributions of mineral and organic soils were shown in Figure 5A,B. Clay contents for mineral and organic soils were skewed forming long tails with only a few samples having values higher than 60%. The average clay content value for organic soils is 15% while for mineral soils is 17%. Organic soils have generally lower clay content as pointed out in Reference [35].

The mean soil reflectance spectra and standard deviations for mineral and organic soils were plotted in Figure 5C,D. The mean spectra of both mineral and organic soils have a similar curve shape whose reflectance values increase with increasing wavelength in the range of 500–1300 nm. The main spectral difference is that the mean reflectance spectrum for mineral soils demonstrates a higher albedo than spectra for organic soils as mineral soils have a lower level of SOC content. It is well known that higher levels of organic material lead to darker soils, and soil reflectance decreases with increasing SOC content especially in the spectral range of 600–750 nm as observed in References [50,70].

The mean soil continuum-removal (CR) spectra and standard deviations for mineral and organic soils were also shown in Figure 5E,F. CR spectra can be used to isolate and identify characteristic absorptions of minerals, organic compounds, and water in soils [8]. Both mineral and organic soils showed absorption peaks near 600, 1400, 1900 and 2300 nm. The absorption depths near 600 and 2300 nm for organic soils are much deeper than mineral soils. The highest correlation between double square-root of the SOC content (SOC1/4) and reflectance is found in the visible region, with a maximum around 600 nm [50]. Around 2300 nm (2309 and 2347 nm) are combinations and overtones of the C-H group, which is characteristic of different organic substances [71]. Mineral soils also have an absorption peak near 2200 nm, which is correlated with clay content [72]. Organic soils have absorption peaks near 1720 nm, which correlated with SOC.

### 3.2. 1D-CNN and Spectral Index for LUCAS Soil Clay Content Estimation

In the architecture of 1D-CNN, four convolutional layers were adopted with weights initialised by a uniform distribution. The optimiser is adamax [73] and loss function is mean squared error (MSE) to train the model. (*R*^2^ = 0.834, *RMSE =* 5.31 and *RPD =* 2.42).

**Figure 6:**
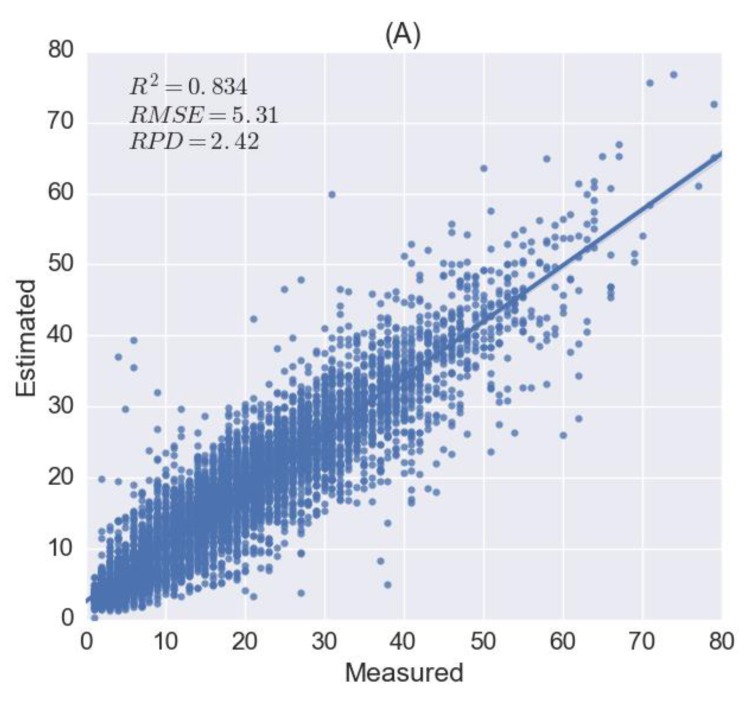

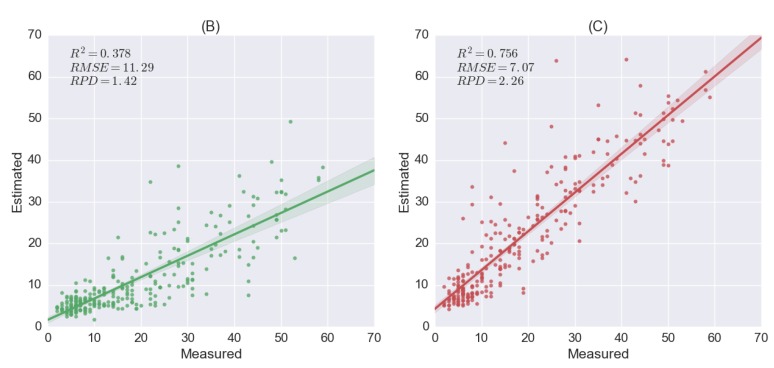
Results of soil clay content estimation for LUCAS mineral and organic soils using one-dimensional convolutional neural network (1D-CNN) and transfer learning. (**A**) The scatter plot of measured and estimated clay content for mineral soils obtained by 1D-CNN model. (**B**) Organic soils using the pre-trained 1D-CNN model developed by mineral soils without fine-tuning. (**C**) Organic soils by fine-tuning the pre-trained 1D-CNN model.

Before fine-tuning the pre-trained 1D-CNN model using organic soils, the number of neurons in the fully-connected layer was reduced from 32 to 16 so as to reduce the training parameters. The result is (*R*^2^ = 0.756, *RMSE =* 7.07 and *RPD =* 2.26). We also tried to directly apply the pre-trained 1D-CNN model without fine-tuning and achieved a comparatively poor accuracy (*R*^2^ = 0.378, *RMSE =* 11.29 and *RPD =* 1.42), as shown in Figure 6B.

**Figure 7:**
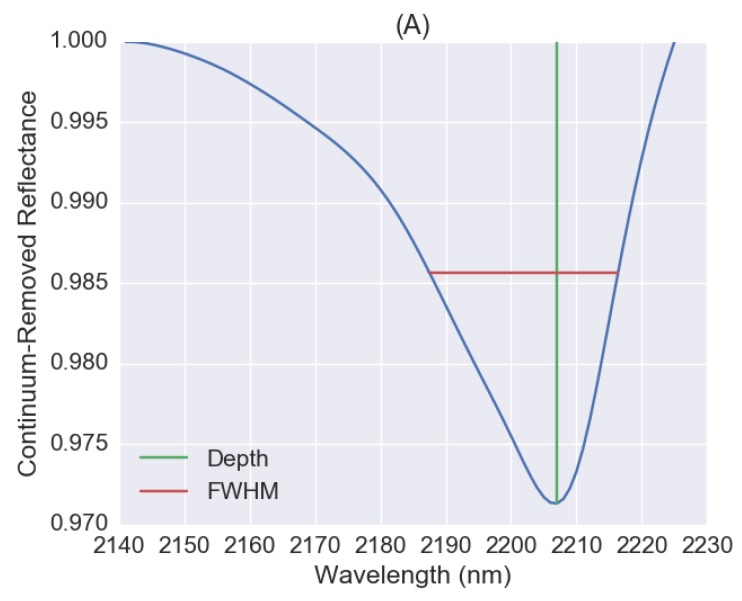

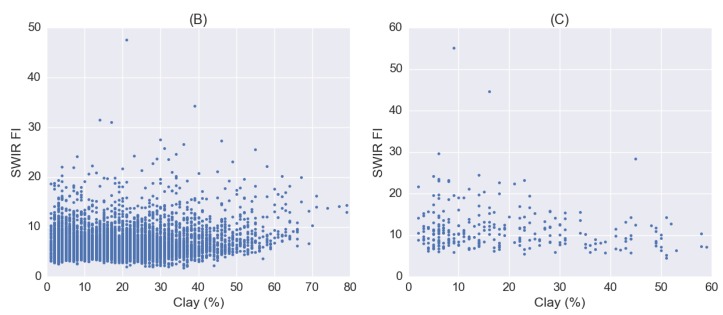
Absorption feature near 2200 nm for the mean spectrum of mineral soils (**A**), and scatter plots between soil clay contents and the corresponding short-wave infrared fine particle index (SWIR FI) values for mineral (**B**), and organic soils (**C**).

The absorption feature near 2200 nm for the mean spectrum of mineral soils was shown in Figure 7A. For mineral, the absorption peak is at 2207 nm, which is very close to 2209 nm as adopted in the spectral index of SWIR FI. The depth is 0.971 and the full-width at half-maximum (FWHM) is 30 nm. However, there is no observed absorption feature near 2200 nm for organic soils. Spectral index failed on both mineral and organic test dataset as shown by the scatter plots between SWIR FI and soil clay content values in Figure 7B,C, especially for soil samples having clay content values greater than 20%. We also tried to adopt the equation for SWIR FI with band combinations at 2207, 2140 and 2225 nm for mineral soils, but did not achieve much improvement. Therefore, we only consider transfer learning based on 1D-CNN for the following application with hyperspectral imagery.

### 3.3. Application of Transfer Learning for Soil Clay Content Mapping Using the Pre-Trained 1D-CNN Model

**Figure 8:**
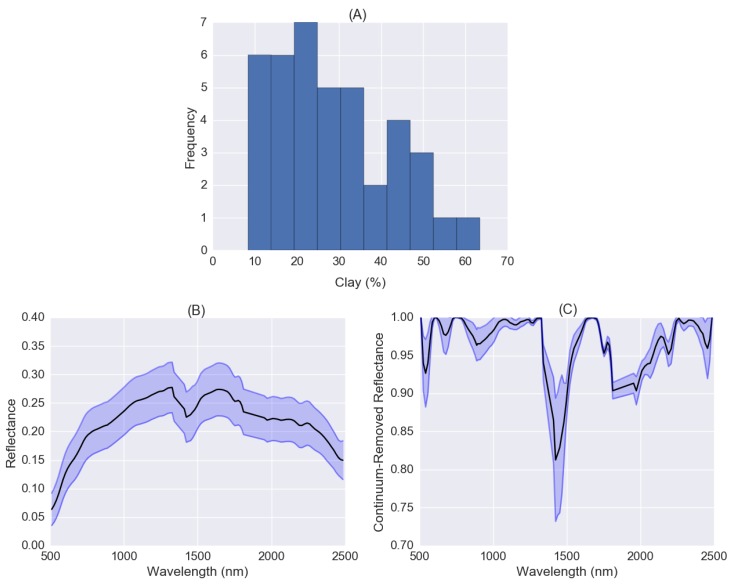
(**A**) Histogram of soil clay content distribution of soil samples collected from study area Cabo de Gata-Nijar; (**B**) mean soil reflectance spectrum and standard deviation derived from the hyperspectral image; (**C**) mean soil continuum-removal spectrum and standard deviation.

The clay content values of field samples vary between 8.4% and 63.4%. The mean soil reflectance spectrum (black line) and standard deviation for spectra extracted from hyperspectral imagery at the locations of field samples were shown in Figure 8B. The overall albedo is lower compared to LUCAS mineral or organic soil spectra measured in the laboratory. The mean soil reflectance spectrum (black line) and standard deviation for spectra for CR spectra were shown in Figure 8C. The absorption depth near 1400 nm is much deeper than LUCAS soil spectra measured in the laboratory, which is caused by water absorption.

**Figure 9:**
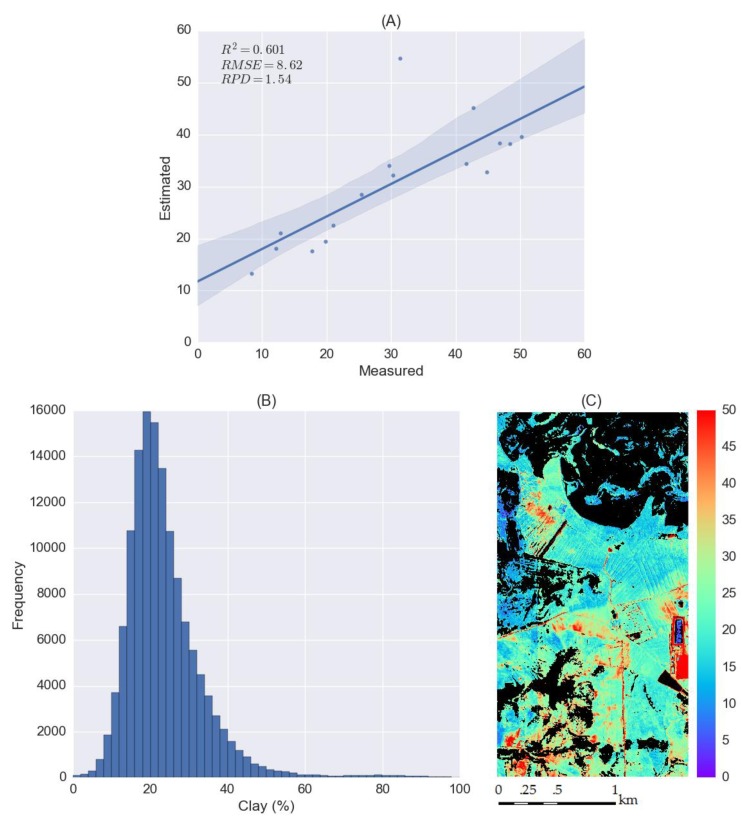
Results of transfer learning for soil clay mapping using hyperspectral imagery and the pre-trained CNN model. (**A**) The scatter plot between measured and estimated clay contents for testing data; (**B**) the histogram of soil clay content distribution of derived soil clay map without considering masked non-bared soil pixels; (**C**) the soil clay map in the study area with masked non-bared soil pixels (black).

The pre-trained CNN model was fine-tuned by 16 field samples collected in the study area and then validated using the other 16 soil samples. The validation accuracy (*R*^2^ = 0.601, *RMSE =* 8.62 and *RPD =* 1.54) was lower than the result obtained from LUCAS organic soils. The fine-tuned model was applied to the whole hyperspectral image except for the non-bared soil pixels. From the histogram of clay content (Figure 9B), it can be seen the distribution of soil clay content was also skewed forming long tails and the majority of soil clay values fell in the range from 10% to 40%. For clay content map (Figure 9C), non-bared soil pixel values were set to 0 and clay content values greater than 50% were set to 50%.

### 3.4. Comparison between Spectral Index and Transfer Learning

Spectral index is a simple and easy implemented algorithm that often only use few bands rather than the full visible near-infrared spectral range. It is particularly efficient in deriving information that relies on the specific spectral response of the targeted object [74]. Although it is suggested that the spectral index is transferable from one sensor to another, SWIR FI proposed in Reference [16], showed little correlation with clay content of LUCAS soils, especially for soil samples having clay content higher than 20%. The absorption peak around 2200 nm for mineral soils is slight different from what observed in Reference [16]. It is pointed out that indices obtained using one instrument could be significantly different from the same indices obtained using other instruments [75]. For organic soils, there is no absorption peak around 2200 nm, because of extremely spectral diverse compared with mineral soils. Besides, Regression models built on one or a few spectral features are often not sufficiently robust for a practical application to a wide variety of soils [43]. Therefore, it is still difficult to use spectral indices for transferable study of quantitative soil properties, especially for different soil categories.

Transfer learning is proposed based on deep learning (DL). With LUCAS mineral soils, the 1D-CNN obtained an accuracy (*R*^2^) of 0.834. Organic and mineral soils from LUCAS data were measured by the same instrument and in well-controlled laboratory. The main difference is the diversity of spectra. For the CNN model, it means the input domain is different. When trying to use the pre-trained 1D-CNN model developed from mineral soils, fine-tuning is required to make the model transferrable from source domain to target domain. By doing that, the *R*^2^ value improved from 0.378 to 0.756. DL provides an end-to-end learning approach with no need for feature engineering. Unlike many prior regression approaches, DL models can be trained on additional data without restarting from scratch, making them viable for continuous learning. Therefore, it is possible to reuse a DL model trained from the large-scale spectral library for local-scale soil property quantification, which makes DL applicable to fairly small datasets. The transferred calibration model obtained an accuracy of 0.601 for soil clay content mapping, which was comparatively lower than achieved by the spectral library. It is pointed out that surface spectral data are generally affected by the confounding effects of soil moisture and soil roughness [76]. Water absorption contributed to the spectral difference between laboratory and airborne hyperspectral data, as shown in Figure 8. Soil moisture has a strong influence on the amount and composition of reflected and emitted energy from the soil surface. Most importantly, water absorption features near 1400 and 1950 nm will mask important spectral information associated with soil variables, including clay [28,77]. A direct standardization (DS) method was proposed to correct the difference between instruments [78] and successfully utilised to reduce the effects of soil moisture and other environmental factors on field Vis–NIR–SWIR spectra [40,79]. For the CNN model, choosing the optimal architecture and training it optimally are still open questions. It is hard to comprehend what is going on under the hood of DL algorithms [80], which could be a problem for non-experts to develop effective DL algorithms or adopt it to different study areas. Besides, it is difficult for CNN to directly incorporate spectral information with other soil properties and location information like support vector machine, random forest and spectrum-based learner, which are very important to improve the estimation accuracies of soil properties. It should be pointed out that the proposed method for soil clay content mapping was only validated on very few samples, because of the limited available dataset, which constrains the generalizability and thus should be further explored by incorporating more soil samples.

### 3.5. Large-Scale Soil Spectral Library for Mapping Soil Properties at the Local Scale Using Hyperspectral Imagery

There are some studies relate to the retrieval of soil properties by taking advantage of large-scale spectral data. The potential of the LUCAS database for the SOC estimation in Belgium and Luxembourg was investigated in Reference [44]. The LUCAS dataset was divided into several classed using a cluster analysis. PLS regression models were calibrated for each class and then adopted to estimate the SOC content on the soil spectra of the calibration datasets of the same class. Soil samples were scanned by the same instrument that used for the LUCAS dataset. The achieved RPD values for the proposed methods were between 1.41 to 2.24. A bottom-up approach was further developed to estimate SOC using hyperspectral imagery [45] and achieved RPD values of 1.7 for Luxembourg data and 1.4 for Belgium data. The PLS regression models developed using the LUCAS dataset were applied to field soil spectra measured in the laboratory instead of hyperspectral imagery. Besides, this approach requires that the large-scale spectral library should contain spectra that closely match those of the local soil samples. For transfer learning, it does not have such a limitation, but it requires a few soil samples to fine-tune the pre-trained model, as demonstrated in the study of transferring the classification model developed using ImageNet to remotely sensed images [60]. The soil clay content map was generated from airborne hyperspectral data by transferring laboratory regression models with methods of model updating, Repfile, Transfer by Orthogonal Projection (TOP) and Piecewise Direct Standardization (PDS) [26]. Transferred models showed better performance than the laboratory model calibrated without transfer. These methods are used to address factors that cause spectral distortions resulting from the different measurement conditions, while transfer learning is a more general approach to develop a transferrable model instead of aiming to solve the spectra standardization problem. However, the above-mentioned methods, including transfer learning are limited to bare fields as the presence of the vegetation may contribute to the spectral confusion with soil reflectance [81]. Spectral mixture analysis was adopted in Reference [43], to extend the mapping capability up to a vegetation coverage of 40% using a feature-based multiple linear regression model.

Soil property model is often calibrated using field samples collected in the same area, which generally yields the best prediction accuracy. This is because the samples used for calibration are geographically close to the target site and thus are expected to have soil properties and spectral responses that are similar to the target samples [19]. However, it often requires large amounts of field work and many hours or days processing the data. It would be great if the model can take advantage of available existing soil libraries. However, it is pointed out that there are still few studies combing the use of laboratory, proximal, and remote spectroscopic sensing research. One reason might be that there are significant challenges posed by the inherent differences between the standardised laboratory measurements and those made under natural conditions [8]. The signal-to-noise ratio of air- or space-borne hyperspectral data is relatively low compared to laboratory data, due to a low integration time over the target area [72]. The application of imaging spectroscopy is also restricted by atmosphere attenuation, revisiting time, sensor radiometric and spatial resolutions, and BRDF effects. While the effort is putting on reducing the effect of water and other environmental factors, the soil community should also be aware of advancements like DL. Although the model for airborne hyperspectral data was less accurate than the laboratory model, it demonstrated the potential of utilising laboratory spectra and hyperspectral imagery for soil property mapping, and it will continuously benefit from the advancement of DL research.

## 4. Conclusions

In this paper, we investigated the potential of using a pre-trained CNN model for the estimation of soil clay content. The success of DL provides a promising approach to mapping soil properties using hyperspectral data with large-scale soil spectral libraries. A 1D-CNN approach was proposed to the estimation of soil clay content and achieved an accuracy (*R*^2^) of 0.834 with LUCAS mineral soil dataset. The 1D-CNN model was further fine-tuned by soil samples collected in the field with spectra extracted from the hyperspectral imagery. The transferred model obtained an accuracy (*R*^2^) of 0.601 for regional soil clay content mapping. To the best of our knowledge, this is the first case study adopting CNN-based transfer learning for soil spectroscopy. However, the proposed approach was tested only on a limited area, and its application to practice is still open, especially to areas with different soil conditions. Besides, the proposed method is limited to bare soils, and the influence of external factors, including vegetation coverage and soil moisture should be further studied. Although the result obtained by the hyperspectral imagery is still not compatible to laboratory spectroscopy, the CNN-based transfer learning provides a new way to make use of both large-scale spectral libraries and hyperspectral data to map soil properties.

## Figures and Tables

**Table 1:** Statistics of soil clay content for the calibration and validation dataset.

Dataset	Number	Mean (%)	Standard Deviation (%)	Min (%)	Max (%)
Calibration	16	30.2	14.1	10.8	63.4
Validation	16	27.7	13.6	8.4	50.2
